# Effect of Oxidative Stress on ABC Transporters: Contribution to Epilepsy Pharmacoresistance

**DOI:** 10.3390/molecules22030365

**Published:** 2017-02-27

**Authors:** Gurpreet Kaur Grewal, Samiksha Kukal, Neha Kanojia, Luciano Saso, Shrikant Kukreti, Ritushree Kukreti

**Affiliations:** 1 Academy of Scientific and Innovative Research (AcSIR), CSIR-Institute of Genomics and Integrative Biology (CSIR-IGIB) Campus, Delhi 110007, India; gpkgrewal@gmail.com (G.K.G.); samkukal07@gmail.com (S.K.); kanojia29.2008@gmail.com (N.K.); 2 Genomics and Molecular Medicine Unit, Institute of Genomics and Integrative Biology (IGIB), Council of Scientific and Industrial Research (CSIR), Mall Road, Delhi 110007, India; 3 Department of Physiology and Pharmacology “Vittorio Erspamer”, Sapienza University of Rome, P. le Aldo Moro 5, 00185 Rome, Italy; luciano.saso@uniroma1.it; 4 Nucleic Acids Research Lab, Department of Chemistry, University of Delhi (North Campus), Delhi 110007, India; shrikant.kukreti6@gmail.com

**Keywords:** epilepsy, ABC transporters, oxidative stress, antiepileptic drugs, pharmacoresistance

## Abstract

Epilepsy is a neurological disorder affecting around 1%–2% of population worldwide and its treatment includes use of antiepileptic drugs to control seizures. Failure to respond to antiepileptic drug therapy is a major clinical problem and over expression of ATP-binding cassette transporters is considered one of the major reasons for pharmacoresistance. In this review, we have summarized the regulation of ABC transporters in response to oxidative stress due to disease and antiepileptic drugs. Further, ketogenic diet and antioxidants were examined for their role in pharmacoresistance. The understanding of signalling pathways and mechanism involved may help in identifying potential therapeutic targets and improving drug response.

## 1. Introduction

Epilepsy is a neurological brain disorder, affecting around 1%–2% of the population worldwide [1]. Its treatment includes use of antiepileptic drugs (AEDs) to control seizures, but 40%–50% of the individuals fail to respond to first line AED monotherapy and 30% are refractory, which puts such patients in life threatening situations and is a major clinical problem [2]. Non responsiveness to antiepileptic therapy is still an unsolved problem, which can be attributed to the intrinsic severity [3], altered drug targets [4], or drug transporter expression [5,6]. A drug efflux transporter hypothesis which is more common talks about pharmacokinetics aspects of the drug resistance [3]. Any drug entering the tissue undergoes three steps of biotransformation: phase 1 (functionalization), phase 2 (conjugation), and phase 3 (excretion). Phase 3, also known as terminal phase of drug metabolism involves the drug efflux transporters, also known as ATP-binding cassette transporters (ABC transporters). ABC transporters are the largest family of transmembrane transporters. There are 48 human transporters grouped in seven families A–G. These transporters are present at different tissue barriers and evolved to protect against various endobiotics and xenobiotics. However, protective functions of ABC transporters interfere with drug efficacy by effluxing drugs out of cells and thus influencing drug pharmacokinetics. The drug transporter hypothesis suggests that overexpression of ABC transporters contributes to non-responsiveness to AED therapy. Various studies have associated ABC transporters such as P-glycoprotein (P-gp, also known as ABCB1], multidrug resistance-associated proteins (MRPs, known as ABCCs), and breast cancer resistance protein (BCRP, known as ABCG2] with AED resistance [6,7].

It is worth noting that the abnormal neuronal discharge occurring in epilepsy consumes massive amounts of energy, accounting for extensive oxidative stress observed in the disease. Hence, oxidative stress has become an emerging event in the pathophysiology of epilepsy. The disturbed pro-oxidant antioxidant balance creates an accumulation of free radicals, which are toxic and can damage lipids, proteins, or nucleic acids and cause mutation and cell death. Therefore, our first goal was to review the contribution of disease and AEDs to oxidative stress. Since ABC transporters are known to be critically involved in influencing drug pharmacokinetics in epilepsy, it becomes important to review the studies showing alteration in ABC transporters in response to oxidative stress, so that we can exploit these studies to understand and comprehend how oxidative stress caused due to disease and drugs can affect ABC transporters. Furthermore, the importance of non-pharmacological treatments like ketogenic diet and antioxidants as an adjunct therapy to the available treatment to target epilepsy is discussed in the review and emphasis on drug-diet and diet-transporter interaction as an essential parameter is also argued.

## 2. Contribution of Epilepsy to Oxidative Stress

Oxidative stress has been implicated in a variety of disorders, including cardiovascular diseases, diabetes, and cancer, but the brain being a most demanding organ for oxygen makes it highly susceptible to oxidative stress. Hence, any pathological condition in brain requiring increased energy consumption will result in elevated reactive oxygen species (ROS) levels [8]. Epilepsy is characterized by neuronal hyperexcitability and requires high energy at the neuronal synapse. This may account for increased oxidative stress levels as a consequence of the disease itself [9].

### 2.1. Patient-Study

Menon et al. performed a study to investigate that increased oxidative stress levels in patients with epilepsy is the result of the disease itself and not due to any of the AED medication. Most studied markers of oxidative stress, i.e., nitric oxide levels; lipid peroxidation; and protein carbonylation (PC) were checked in serum. The results showed a significant increase in levels of malondialdehyde (MDA, an index of extracellular lipid peroxidation) and PC in patients with epilepsy as compared to controls. However, AED treatment revealed no difference [10]. In a study, Lopez et al. compared the redox status of drug-resistant patients with temporal lobe epilepsy. Blood markers of oxidative damage were found to be increased in patients [11]. On similar lines, Rumia et al. performed experiments in cortical samples of patients. Neocortical samples taken from drug resistant patients with epilepsy were checked for oxidative stress markers and compared with non-epileptic group. Levels of 8-Oxo-DG (a major product of DNA oxidation) and superoxide anion (O_2_^−^) were significantly higher in the patient group. Moreover, activity of catalase which occurs in response to scavenge free radicals was also elevated. Since patients were on AEDs in both of these studies, it cannot be concluded that the increase was solely the consequence of epilepsy [12].

### 2.2. In Vivo Studies

Kainic acid, which is well known to induce seizures in vivo, was administered to Sprague-Dawley adult rats. Increase in activity of superoxide dismutase (SOD), catalase, and glutathione peroxidase (GPx) was observed within five days of exposure. Effect on lipid and protein oxidation was also seen as early as 8 h [13]. Another study aimed to find out the cellular compartment responsible for producing free radicals in epileptic brain, again involved the use of kainite model. Both cytosolic and mitochondrial aconitase enzyme are known to be the extremely sensitive markers of oxidative stress induced inactivation. Inactivation of this enzyme in kainate induced rat hippocampal mitochondrial fraction and not in cytosolic fraction, revealed mitochondrial produced ROS as the causal factor for neuronal loss observed in epilepsy [14]. The same group, in 2005, further elucidated that O_2_^−^ ions are also produced extracellularly as a result of seizure-induced hippocampal damage, possibly through activation of brain NADPH oxidase activity [15]. Another interesting in vivo examination done in 2013 aimed at direct measurement of free radicals formed in brain tissue in epileptic condition. Using electron paramagnetic resonance spectroscopy targeted at brain tissues of male Wistar rats, investigators compared free radical formation in rats, which were induced for seizure by convulsant flurothyl and non-induced control rats. After 60 min of induction, hydroxyl and nitroxyl radicals were significantly higher in the experimental group, which was reversed after administering potent antioxidant melatonin [9]. Similar types of studies have also been performed in other epileptic models such as electrical kindling [16], pilocarpine-induced [17], etc., suggesting production of free radicals in seizures. Epilepsy induced oxidative stress can elicit a chain reaction, causing extensive neuronal damage in the brain, further aggravating epileptic seizures.

Epilepsy can also be a consequence of oxidative stress. Since the maximal contribution of cellular ROS comes from a leaky electron transport chain of oxidative phosphorylation in mitochondria [18], defects in mitochondrial respiratory chain enzymes suggests oxidative stress can also be the cause of increased seizure activity. This was indicated in an exciting study in which brain specimens of 57 therapy-resistant temporal lobe epilepsy patients undergoing surgery were taken. Activity of the two key respiratory chain enzymes NADH:CoQ_1_ oxidoreductase (complex I) and cytochrome c oxidase (complex IV) were measured. Complex 1 activity was found to be decreased, which was the possible reason for enhanced neuronal cell loss and vulnerability to seizures [19]. Also, a known form of epilepsy known as myoclonic epilepsy and ragged-red fiber disease (MERRF) is associated with mitochondrial tRNA mutation [20]. Again, in an attempt to study the role of mitochondrial oxidative stress to be involved in epilepsy pathogenesis, Liang at al. (2012), generated SOD2^−/−^ mice, lacking an important mitochondrial antioxidant superoxide dismutase 2. EEG monitoring revealed an increased frequency of motor seizure activity in these mice with elevated brain levels of oxidative stress as confirmed by the levels of aconitase, coenzyme A (CoASH), and its disulphide (CoASSG) and 3-nitrotyrosine. This observation was reversed when endogenous ROS generation inhibitor AEOL 11207 (lipophilic metalloporphyrin) was administered to SOD2 deficient rats [21].

## 3. Antiepileptic Drugs and Oxidative Stress

There are a number of indications which reveal that, apart from contribution of disease, AEDs can also modulate pro-oxidant/antioxidant balance. Experimental investigations for the effect of AEDs on oxidative stress has been done both at in vitro and in vivo levels.

### 3.1. In Vivo Studies

Willmore et al. (1984), reported that phenytoin (PHT) treatment in rats prevented the occurrence of convulsive and EEG seizures; however, lipid peroxidation was unaffected. This revealed PHT masked convulsive seizures without preventing peroxidation causing biochemical brain injury [22]. Mahle and Dasgupta (1997) observed elevated concentrations of lipid hydroperoxide and reduced levels of antioxidant capacity of the sera of patients with epilepsy receiving PHT as compared to controls [23]. Another patient study investigated the levels of the serum MDA, serum copper, serum zinc, copper/zinc SOD, and reduced glutathione (GSH) concentrations in female patients with epilepsy on PHT monotherapy and confirmed the increase in oxidative stress [24]. Another group studied the effect of PHT on male Wistar rats and reported increase in MDA levels and reduction of GSH levels in the brain indicating towards oxidative stress [25]. Oxidative stress has also been observed in fish brain on long term exposure to carbamazepine (CBZ) [26]. However, Yuksel et al. (2001) studied the effects of valproate (VPA) and CBZ on children with epilepsy and concluded that antioxidant systems were better regulated in patients on CBZ as compared to VPA [27]. Similarly, another study reported higher oxidative stress in children with epilepsy on VPA [28]. Out of the conventional first line AEDs, CBZ was found to be better antiepileptic for the control of free radical-related seizures and trace element levels were better maintained with CBZ than with VPA and PHT therapies [29].

Studies with second line AEDs have also investigated their role in oxidative stress. Reduced antioxidant capacity and toxic liver dysfunction have been observed in rats on topiramate for three months [30]. However, a study by another group observed significant reduction in kainate produced lipid peroxidation [31]. Anti-oxidizing and the neuroprotective role of levetiracetam (LEV) has been identified in mice administered pilocarpine after LEV [32]. The neuroprotective role of Zonisamide (ZNS) has been found in a kainate convulsion model in rats and iron-induced epileptogenic foci in the rat brain [33].

### 3.2. In Vitro Studies

Hepatotoxicity with aromatic AED viz. CBZ, PHT, and phenobarbital in rat liver microsomes was reported and it was suggested that it might be mediated by the oxidative stress induced by the drug metabolites [34]. A group investigated the effect of AEDs on oxidative stress by studying various parameters like: lactate dehydrogenase (LDH) and glutamine synthetase (GS) levels, ROS production, lipid peroxidation, and DNA fragmentation in an astrocyte culture from rats. CBZ, topiramate, and oxcarbazepine caused oxygen stress, whereas gabapentin, LEV, LTG, tiagabine, and ZNS produced no significant changes [35]. Oliveira et al. demonstrated neuroprotective and antioxidant effect of LEV and clonazepam in mice brain homogenate by preventing pro-oxidant changes, reducing lipid peroxidation, nitrite-nitrate content, and catalase activity and increasing GSH levels [36]. LEV has potential for treating oxidative stress and inflammation in the peripheral nerves [37]. The protective role of ZNS on GSH levels in astroglial C6 cells [38] and reduced lipid peroxidation and cytosolic-free Ca^2+^ in 1-Methyl-4-phenylpyridinium (MPP+) model of Parkinson’s in neuronal PC12 cells [39] were observed.

The generated free radicals from AEDs cause toxicity by binding to fundamental biomolecules of cells and causing cell injury and death rather than having a neuroprotective effect on brain cells, aggravating the disease state. Thus, it has been observed from literature that conventional AEDs cause more oxidative stress and free radical generation than newer AEDs.

## 4. Oxidative Stress Regulates ABC Transporters

ABC transporters have evolved to protect against oxidative stress generated from various reactive oxygen species. Toxic compounds generated are eliminated by ABC transporters after they are detoxified by conjugation to GSH, glucuronide, and sulphate. However, the protective function of ABC transporters may interfere with drug retention leading to pharmacoresistance through their efflux.

P-gp, MRPs and BCRP transporters have been associated with AED resistance. P-gp is the extensively studied transporter in epilepsy followed by multidrug resistance related proteins (MRPs, MRP1–5) and breast cancer related protein (BCRP; ABCG2). A first study by Tishler et al. that led to the formulation of multidrug transporter hypothesis showed increased expression of P-gp in capillary endothelial cells of patients with medically intractable epilepsy. They also reported decrease in steady state intracellular concentrations of PHT in P-gp expressing neuroectodermal cells compared with P-gp negative cells [40]. Further, several studies reported the overexpression of P-gp, MRPs (MRP1, 2, and 5), and BCRP in different epilepsy animal model studies as well in patients with pharmacoresistant epilepsy [41,42,43,44,45,46,47,48,49]. These transporters (P-gp; MRP1, 2, 5; BCRP) are localized at the apical side of brain capillary endothelial cells, which is an apt position to interfere with drug efficacy by effluxing it back to the bloodstream [50]. However, localization of MRP1 and MRP5 has also been reported on the basolateral side of endothelial cells, indicating a negative role in drug resistance [51]. So, further studies are needed to confirm their localization and role.

The efflux transporters remove their substrate drugs and restrict brain uptake. So, it is important to understand the substrate relationship of AEDs with ABC transporters. Lamotrigine, oxacarbamazepine, phenobarbital, and PHT are definite substrates of P-gp [52]. Evidence from various in vitro and in vivo studies indicate that CBZ is a substrate of P-gp and MRP2 [52,53,54,55,56,57] while several reports refute this [58,59,60,61,62]. There are fewer studies of CBZ with BCRP and it has been found by accumulation assays that there is no interaction of CBZ with BCRP [63]. In vitro and in vivo studies have been discussed to understand the relationship of CBZ with P-gp and MRP2. A study in LLC-PK1 cells transfected with MDR1 and primary porcine brain capillary endothelial cells demonstrated by uptake assay using calcein acetoxymethlyester that CBZ inhibited P-gp efflux function [53] indicating its substrate relationship. Whereas another study using rhodamine123 (Rho123) as substrate in OS2.4/Doxo cells (canine osteosarcoma cells induced via exposure to doxorubicin) concluded CBZ did not affect uptake of Rho123 and not a substrate of P-gp [58]. CBZ was not found to be a substrate of P-gp by concentration equilibrium transport assay in the LLC and MDCKII monolayer models [59]. However, a major active metabolite of CBZ, carbamazepine-10,11-epoxide was a substrate of P-gp [64]. CBZ was found to be a substrate of MRP2 using membrane vesicles expressing MRP2 and 5,6-carboxyfluorescein substrate in HEK239T cells [54]. Whereas Radisch et al. (2014) suggested no substrate relationship of CBZ with MRP2 using in vitro approach [60]. Studies of MRPs in cell lines revealed none of AEDS being substrate of human MRP1, 2, or 5. However, authors acknowledge that in vitro assays may produce false negative results [61].

There are also in vivo studies to study substrate relationships. By using in vivo microdialysis in rats, Potschka et al. (2001) found enhanced concentration of CBZ in the extracellular fluid of the cerebral cortex in the presence of P-gp and MRP inhibitor verapamil and probenecid respectively. The data indicated that both P-gp and MRP participate in the regulation of extracellular brain concentrations of the CBZ [55]. There are also in vivo studies using P-gp knockout mice mdr1a(−/−) and mdr1a/1b(−/−). Sills et al. (2002) found higher brain/serum concentrations ratios of CBZ in knockout mice than wild type mice. Thus indicating a substrate relationship with P-gp [56]. However, another study using mdr1a/1b(−/−) mice did not find significant difference in CBZ concentrations [62]. A study in patients with intractable epilepsy found that extracellular fluid concentrations of CBZ were significantly lower than their cerebrospinal fluid concentrations [57]. According to a study by Zhang et al. (2012), CBZ is a possible substrate of P-gp because human data evidence is positive, but rat models and cellular model evidence are negative [52]. To reach to concrete solution, there is need of further in vivo studies, including PET with labelled MRP and P-gp substrate. Thus, these studies depict the importance of ABC transporters in drug disposition. Hence, we have further reviewed the studies where ABC transporters are regulated by oxidative stress.

As discussed earlier, wherein reactive oxygen species are involved in cytotoxicity, they can also play important role in signal transduction [65] through various transcriptional factors, hypoxia-inducible factor-1(HIF-1), Nuclear factor-κB (NF-κB), and Nuclear factor E2-related factor-2 (Nrf2). In turn, these transcription factors can regulate expression of ABC transporters which can interfere with drug efficacy. NF-κB activation is involved in the seizure susceptibility and seizure induced brain P-gp overexpression in rats [66]. Nrf2 is a transcription factor which is a cellular sensor for oxidative stress. Maher et al. demonstrated that hepatic expression of Mrp2, Mrp3, and Mrp4 can be induced in response to oxidative conditions and treatment with Nrf2 activators [67]. The author has suggested that Nrf2-mediated regulation of Mrps is a crucial mechanism for hepatic transport as well as for treatment of patients with liver diseases having oxidative stress. The role of Nrf2 was further explored by another group in the blood–brain and blood–spinal cord barrier. It was demonstrated on activation of Nrf2 in response to oxidative stress protein expression and activity of P-gp, Bcrp, and Mrp2 were increased. This signalling involved the role of p53, p38, and NF-κB [68]. Thus, the blood-CNS barriers are tightened by oxidative stress to provide neuroprotection, but that led to reduced penetration of drugs.

Ronaldson et al. in an attempt to demonstrate the role of MRPs in regulating oxidative stress observed in brains of HIV-1 infected patients found that these MRPs are also in turn regulated by these molecules. Primary rat cultured astrocytes were treated with HIV-1 gp120. Significant increase in cellular ROS and GSSH/GSH ratio was found at 24 h, which was indicative of oxidative stress. Besides, MRP1 mRNA and protein expression also showed a significant increase. To elucidate that oxidative stress is involved in this increase, the same cells were exposed to an H_2_O_2_ generating system. Surprisingly, MRP1 protein expression increased 2.5-fold after 90 min of exposure, along with the increase in its functional activity [69].

Upregulation of P-gp and other ABC transporters including MRP1, 2, 4, and BCRP protein expression by an air pollutant diesel exhaust particle (DEP), affecting CNS pathology, provides another compelling indicator of oxidative stress mediated regulation of ABC transporters. DEP is shown to generate ROS through NADPH oxidase activation. Blockage of NADPH oxidase prevented DEP mediated increase in P-gp in isolated rat brain capillaries. When tissue culture medium was supplemented with SOD and catalase as ROS scavengers, again P-gp activity decreased. This suggests the role of oxidative molecules in transporter regulation [70]. Ammonia, an important neurotoxin involved in hepatic encephalopathy, is known to induce oxidative stress in the brain. In a study by Zhang et al. 2015, hyperammonemic rats were used to check the effect of ammonia on ABC transporters at blood brain barrier (BBB) [71]. It was observed that function of P-gp and Mrp2 was elevated at 6 h. This was accompanied with an increase in levels of NO and MDA with a decrease in SOD activity. NF-κB activation was found responsible for transporter upregulation. Further, another recent report from the same group, concluded that ROS mediated ERK1/2 phosphorylation was involved in the downregulating BCRP expression in rat BBB [72].

Seebacher et al. 2015, in its study depicted how increased ROS levels contribute to MDR phenotype. Lung carcinoma cell lines as a tumor model were used to validate this. When the cells were exposed to varying glucose conditions, ROS generation increased in response to high glucose as well as glucose deprived conditions. Both these cases were also accompanied with simultaneous increase in P-gp activity. ROS induced P-gp activity was confirmed by reversal of increased P-gp activity after treatment with antioxidant *N*-acetylcysteine (NAC). As previously mentioned, again NF-κB was established to be the mediator [73]. Butylated hydroxyanisole (BHA) which is a phenolic antioxidant was shown to induce gene expression of MRP1 transporter in rat liver [74]. Thus, it could be concluded that not only the oxidative stress molecules, but the antioxidant defense system elicited as a counter response, can also alter expression of efflux transporters.

The literature on the direct involvement of oxidative stress and ABC transporters in epilepsy is scarce. Zhang et al. reported increased P-gp expression in astrocytes of the hippocampus in rats after intracerebroventricular kainate injections and concluded that the increase in expression could be part of a cellular stress response program in these cells induced after neuronal injury [41]. Further, Rizzi et al. reported reduced brain/plasma concentration of PHT in kainite model at the time of maximum induction of P-gp. Thus, seizures induced P-gp expression changes affect PHT concentrations in the brain and contribute to pharmacoresistance [75]. Along similar lines, another study observed the overexpression of P-gp in hippocampal neurons of pilocarpine- and kainate-treated animals and related it to protective response to neurotoxic compounds [76]. Another study was performed on MRPs other than P-gp. Electrically induced status epilepticus in rats resulted in elevated expression of MRP1, MRP2, and BCRP in parahippocampal cortex blood vessels and surrounding astrocytes. This increase was associated with lower brain PHT levels compared to control rats and which was reversed by MRP inhibitor probenecid. The authors suggested cellular stress caused by seizure as a possible reason for the induction [48].

There are other studies linking inflammation (which is elicited in response to oxidative damage) with ABC transporters [77,78,79]. More studies are warranted in this area to identify signalling molecules affecting expression of ABC transporters contributing to drug resistance. NF-κB emerged as a promising target that could be targeted to increase the efficacy of AEDs to overcome pharmacoresistance [66].

## 5. Role of Ketogenic Diet and Antioxidants in Pharmacoresistance

Managing patients with pharmacoresistance epilepsy is a challenge and requires a structural multidisciplinary approach. Treatment is limited to surgery and aggressive combination treatment with available AEDs. Non-pharmacological alternative treatments like implementation of ketogenic diet and antioxidants, for the symptomatic treatment of epilepsy have been very promising in recent times.

Ketogenic diet is a medically regimented, high-fat, low protein/carbohydrate diet, used to treat pediatric and refractory epilepsy. The ketogenic diet is also an important coadjuvant treatment for most refractory and generalized epilepsies—such as Dravet, Doose, Lennox-Gastaut and West syndromes [80]—and is often used as an adjuvant to radiation and chemotherapy to treat various malignant gliomas [81,82]. Several clinical studies have also raised the possibility that it may confer long lasting therapeutic benefits for patients with epilepsy and also in a broad range of brain disorders characterized by the death of neurons [83,84,85]. Such dietary therapies focussing on caloric restriction and fasting have shown to cause metabolic changes by increasing the level of ketone bodies (principally β-hydroxybutyrate (BHB), acetoacetate (ACA), and acetone) in blood and lowering the glucose levels [86]. A microarray study by Bough et al. 2006 has shown that the ketogenic diet causes coordinated upregulation of hippocampal genes encoding energy metabolism and mitochondrial enzymes [36]. Ketone bodies are found to influence ROS generation by decreasing free radical generation by various mechanisms such as by reducing the amount of coenzyme Q semiquinone [43], increasing glutathione peroxidase activity [44], and increasing production of mitochondrial uncoupling proteins [45,46]. Thus, ketogenic diet modulates mitochondrial energy production by increasing mitochondrial antioxidant status and thereby controls seizure frequency in patients on the diet [47]. Polyunsaturated fatty acids (PUFAs) from ketogenic diet have known to suppress fast, voltage-gated sodium channels [87] and L type calcium channels [88] both in cardiac myocytes and hippocampal neurons [89]. Elevation of arachidonic acid and docosa-hexanoic acid in serum has been corresponding to seizure control during ketogenic diet has been observed in clinical studies [80]. However, these observations are inconsistent [90].

In addition, it has been speculated that PUFA exert their diverse effects on ion channels and by activating nuclear receptors such as peroxisome proliferator-activated receptor-alpha (PPARα) [91,92] that regulates the transcription of numerous genes linked to energy, amino acids, and neurotransmitter metabolism. PPARα activation induces several oxidation enzymes, resulting in oxidative stress, which in turn is prevented by Nrf2, by mediating the induction of xenobiotic efflux transporter genes, namely multidrug resistance–associated protein (Mrp) transporters (Mrp3 and Mrp4] in mice on PPARα agonist (fatty acids) administration [93]. Furthermore, Nrf2 activation with sulforaphane in vivo or in vitro, both in blood–brain and blood–spinal cord barriers, has shown an increase in expression and transport activity of ABC transporter pumps (abcb1, abcc2, and abcg2) [68]. In addition, fasting associated fatty acid release from adipose tissues is found to alter hepatic transporter genes—namely abcb4, abca1, abcg5 and abcg8 expression—in a PPARα dependent manner [94]. To reduce the oxidative damage, PUFAs increase ABC transporter expression acting as neuroprotectants but this adaptive response may interfere with drug efficacy. We contemplated the effect of a ketogenic diet on efflux transporters as they are seen to be the key players in evoking pharmacoresistance. We observed that research studies pinpointing the role of ketogenic diet in altering efflux transporters expression are underdetermined. The extent and degree of efflux transporters expression contributing to pharmacoresistant epilepsy by ketogenic diet is still unclear and further studies are warranted.

The burgeoning evidence from recent research studies has considerably highlighted the key role of the antioxidant/oxidative system in the pathogenesis of epilepsy. Antioxidants, endogenously formed and exogenously administered such as lipoic acid, mexidol, tocopherol, melatonin, resveratrol, vitamin C, and vitamin E have been evaluated for their efficacy in various models of seizures and shown to protect the brain against seizure induced damage as well [48,49,50]. The neuroprotective effect of antioxidants has been confirmed by decreased levels of oxidative markers and an increased level of enzymatic and non-enzymatic antioxidant enzymes in various models of seizures and epilepsy. A study demonstrated the dose dependent anticonvulsant role of NAC in pentylenetetrazole-induced seizures in mice and is suggested to have potential for use in the absence of seizures in humans [95]. NAC is also reported to reverse the ROS induced P-gp expression [73] indicating it could be explored as a promising target for controlling pharmacoresistance in epilepsy, but exceptions have also been observed [96,97].

Though dietary supplements can have neuroprotective effects, concomitant treatment with AEDs can result in drug-diet interactions, further resulting in change in pharmacokinetic and pharmacodynamic parameters. Induction of CYP3A4 has been observed with Vitamin E [98] treatment resulting in reduced concentration of its substrate such as CBZ, PHT, and phenobarbital [99]. Besides, CYP3A4 can be inhibited by resveratrol and the increase in CBZ levels in rats [99]. As discussed, earlier ketogenic diet can result in altered expression of ABC transporters that would further lead to altered drug efficacy and drug toxicity. Thus, it is imperative to monitor the inclusion of diet supplements with AED treatment to improve quality of life of epilepsy patients without potential clinical implications.

## 6. Conclusions

**Figure 1:**
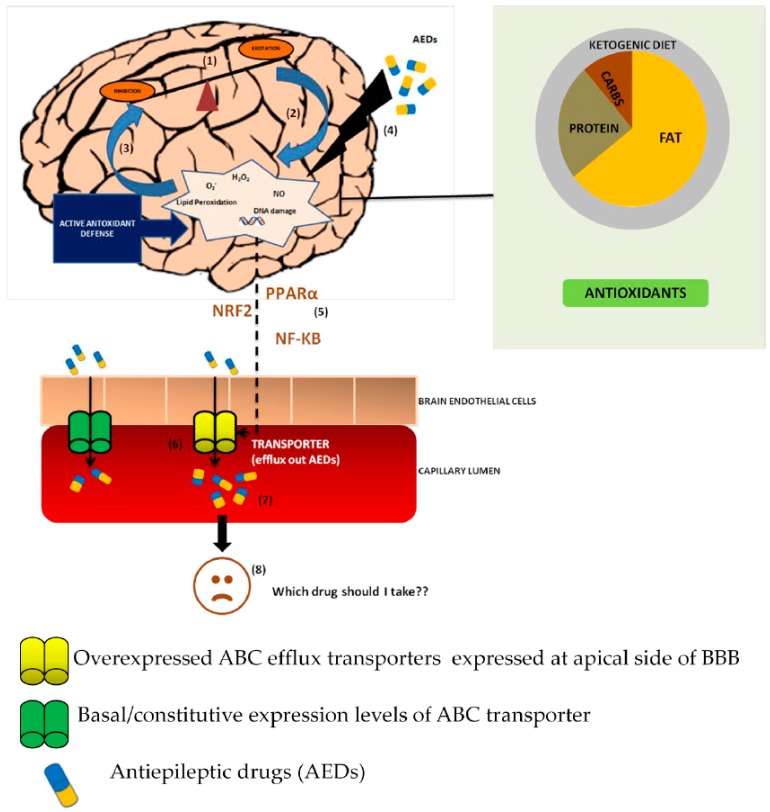
Modulation of ABC efflux transporter at blood brain barrier (BBB) by oxidative stress: possible mechanism of pharmacoresistance in epilepsy. Epilepsy results when the balance between neuronal excitability and inhibition is tipped towards excitability (**1**); This imbalance increases production of brain ROS molecules, leading to oxidative stress (**2**); The elevated stress in turn aggravates the epileptic condition forming a vicious cycle (**3**); First line AEDs given as a treatment to patients with epilepsy also raises oxidative stress levels, which become undesirable for the patients (**4**); This oxidative stress through activation of various signaling molecules such as Nrf2, NF-KB, PPARα etc. (**5**) alters the basal transporter expression (**6**); The resulting consequence is increased efflux of AEDs from brain (**7**); which are substrate of these transporters resulting in pharmacoresistant phenotype (**8**). To address the problem of oxidative stress damage, antioxidants as an adjunct therapy along with AEDs and ketogenic diet seem promising as efficient neuroprotectants. However, their role in pharmacoresistance is still unclear.

Reports suggest that epilepsy is well-associated with oxidative stress levels in brain causing extensive neuronal damage. There is an extensive antioxidant imbalance and increased production of reactive species in epilepsy patients. Older generation AEDs cause oxidative stress and impact life quality of patients with epilepsy as compared to newer AEDs. Moreover, even after the development of new generation AEDs, 30% of the patients still remain refractory. There is a considerable gap in understanding this pharmacoresistance. Since overexpression of ABC efflux transporters plays a critical role in limiting access to AEDs and epilepsy drug resistance, the fact that oxidative stress generated during the course of the disease or due to AED treatment alters transporter expression at the blood-brain barrier. This may result in further worsening situation of patient with epilepsy. The alteration may result in either overexpression of transporters contributing to efflux of AEDs or downregulation culminating in increased cellular toxicity due to reduced efflux of oxidative molecules. From the above studies, we can conclude that oxidative stress activates a series of downstream factors or proteins—including Nrf2 and NF-κB—to finally regulate ABC transporters, thus reducing the brain drug efficacy (Figure 1). Identification of potential therapeutic targets and designing of molecules that can target stress induced signalling can help to enhance drug response and reduce disease severity. Further, the use of antioxidants along with AEDs could help in neuroprotective and antioxidative effect. The use of antioxidants as additional therapy seems promising to reduce neural damage and improve response. Further studies are needed to optimize therapy and avoiding drug-diet interactions for encouraging clinical results.
